# Extent of microbial over-identification of endotracheal aspirate versus bronchoalveolar lavage in the diagnosis of ventilator-associated pneumonia

**DOI:** 10.1186/s13054-024-04931-1

**Published:** 2024-05-09

**Authors:** Alessandra Bisanti, Valentina Giammatteo, Giuseppe Bello, Domenico Luca Grieco, Gennaro De Pascale, Massimo Antonelli

**Affiliations:** 1 Department of Emergency, Intensive Care Medicine and Anesthesia, Fondazione Policlinico Universitario A. Gemelli IRCCS, Università Cattolica del Sacro Cuore, L.go A. Gemelli 8, 00168 Rome, Italy; 2 Istituto di Anestesiologia e Rianimazione, Università Cattolica del Sacro Cuore, Rome, Italy

Respiratory tract secretion sampling is widely used in intensive care units (ICUs) for the microbiological diagnosis of ventilator-associated pneumonia (VAP). However, a consensus on the optimal method for microbiological confirmation of clinically suspected VAP is still lacking [1–3]. Therefore, sampling methods are commonly selected depending on operator expertise, costs, and personal preference.

It is generally accepted that in patients with suspected VAP, endotracheal aspirate (ETA) collection leads to an over-identification of bacteria, which would result in an inappropriate/excessive antibiotic usage compared with distal airway samples [1]. However, there is no convincing evidence to support this assumption.

In a previous clinical study, we demonstrated that cultures of BAL samples taken from the right and left lungs yield discordant results in approximately 40% of cases, reflecting the limited repeatability (75%) of quantitative BAL cultures when BAL is performed in two adjacent segments of the same lung lobe [4].

Given this wide variability in microbiological results by sampling different lung sites, we cannot exclude that a more proximal collection of respiratory tract secretions may comprise the microbiological flora of multiple distal areas, rather than being at risk for redundant and inappropriate over-identification of germs.

While several clinical studies [1–3] have compared the clinical role of ETA and BAL in the diagnosis of VAP, data on the comparison between the microbial growth in ETA and BAL cultures, in particular regarding the potential over-identification, are scarce. Therefore, we carried out an analysis of data provided by the database of a previous study [5] conducted on microbiological surveillance in mechanically ventilated patients.

During a one-year prospective observational study, we enrolled all consecutive adult patients on invasive mechanical ventilation for at least 48 h in a 20-bed general ICU. Patients underwent subglottic secretion and ETA surveillance cultures twice weekly (211 sample pairs), and blind BAL in case of clinical suspicion of pneumonia.

In the present analysis, the primary endpoint was to determine the rate of microbial over-identification of pathogens isolated in ETA cultures with respect to BAL cultures in mechanically ventilated patients with the clinical suspicion of VAP. Over-identification of ETA culture was established when at least one pathogen isolated in the ETA was not found in the BAL fluid.

Following a clinical suspicion of VAP, 44 BAL cultures were obtained from 40 patients. Microbiological confirmation of pneumonia was achieved in 27 (61%) of these BALs.

Among 44 clinically suspected VAPs, full microbiological concordance between ETA and BAL culture results was observed in 33 cases (75%) (Fig. 1A). Episodes with microbiological discordance of ETA and BAL sample pairs included partial (n = 7) and total (n = 4) disagreement of culture results. There was no significant association between ETA/BAL microbiological concordance and clinical variables, such as mechanical ventilation data, laboratory tests, and antimicrobial therapy at the time of sampling. No significant association was found between microbiological concordance and BAL fluid bacterial burden.

**Fig. 1:**
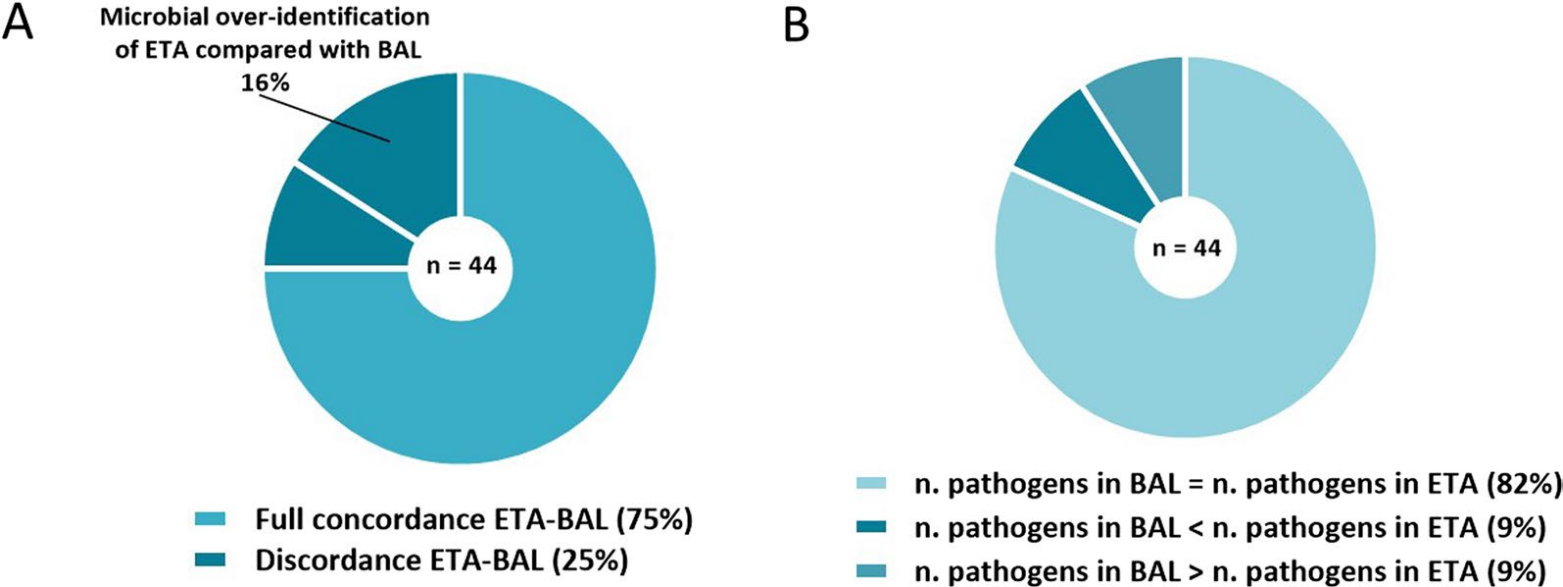
**Detailed representation of ETA and BAL culture results**. *BAL* bronchoalveolar lavage, *ETA* endotracheal aspirate. Among 178 patients intubated in the intensive care unit during the study period, 138 were mechanically ventilated for at least 48 h and 109 were enrolled. Forty-four episodes of clinically suspected ventilator-associated pneumonia were analyzed. Microbiological confirmation of pneumonia was achieved in 61% of cases. In microbiologically discordant ETA/BAL sample pairs, the number of pathogens identified by culture results ranged from 0 to 2 and from 0 to 3 in ETA and BAL fluid, respectively. Over-identification of ETA culture was established when at least one pathogen isolated in the ETA was not found in the BAL fluid.

Using BAL culture as the reference standard of VAP, microbial over-identification of ETA culture was 16%. By the analysis of microbial flora isolated in ETA and BAL sample pairs, the raw number of pathogens isolated in ETA cultures was equal to the number of pathogens isolated in the respective BAL cultures in 36 episodes (82%); the number of episodes where the pathogen count was higher in ETA versus BAL (n = 4 (9%)) was equal to the number of episodes with a higher pathogen count in BAL versus ETA (n = 4 (9%)) (Fig. 1B).

Based on our findings, whether ETA cultures entail microbial over-identification with respect to (single-site) BAL is poorly supported. Moreover, even by assessing the risk for excessive antimicrobial therapy solely according to the raw number of pathogens isolated in ETA and BAL sample pairs, this risk would be equally low regardless of the technique used. Furthermore, we cannot exclude that even if fewer pathogens were detected in BAL compared with ETA cultures, the reduced use of antibiotics might stem primarily from targeting fewer microorganisms rather than targeting those actually responsible for the pneumonia.

The main question in this context is which reference standard technique should be used for making diagnosis of pneumonia. Furthermore, as ETA culture has been found to have a higher sensitivity and a poorer specificity compared to BAL culture in a large meta-analysis [3], another central question is whether false negatives, which would result from low sensitivity (and are potentially associated with insufficient therapy), have more or less dangerous effects than false positives, which would result from low specificity (and are potentially associated with excessive/unnecessary therapy).

Concerning diagnostic bronchoscopy, the early recommendation of using the bronchoscope to collect lower respiratory tract specimens from the site of greatest radiographic abnormality has been questioned for several years now, as findings on the role of chest X-ray in predicting the presence of VAP have shown limited accuracy [4]. Instead, bronchoscopy should probably be considered in particular situations, such as excavated lung lesions, lung abscess or risk for tracheo-bronchial lesions.

In conclusion, we found no evidence that ETA over-identifies pathogens compared to (single site) BAL in patients suspected of having VAP.

## Data Availability

The datasets generated and/or analyzed during the current study are not publicly available but are available from the corresponding author on reasonable request.

## Abbreviations

BAL: Bronchoalveolar lavage
ETA: Endotracheal aspirate
ICU: Intensive care unit
VAP: Ventilator-associated pneumonia
